# Association of *PXR* and *CAR* Polymorphisms and Antituberculosis Drug-Induced Hepatotoxicity

**DOI:** 10.1038/s41598-018-38452-z

**Published:** 2019-02-18

**Authors:** Yu Wang, Xi Xiang, Wei-Wei Huang, Andrew J Sandford, Shou-Quan Wu, Miao-Miao Zhang, Ming-Gui Wang, Guo Chen, Jian-Qing He

**Affiliations:** 1 Department of Respiratory and Critical Care Medicine, West China Hospital, Sichuan University, Chengdu, Sichuan China; 2 Centre for Heart Lung Innovation, University of British Columbia and St. Paul’s Hospital, Vancouver, BC Canada; 3 Division of Geriatrics, Sichuan Provincial People’s Hospital, Chengdu, Sichuan China

## Abstract

A combination therapy of multiple drugs including isoniazid, rifampicin, ethambutol and pyrazinamide has been proven to be an effective option for the vast majority of tuberculosis (TB) patients. However, various adverse drug reactions (ADRs) limit its merit, with anti-TB drug-induced hepatotoxicity (ATDH) being a common and sometimes severe ADR. This study aimed to investigate the association between polymorphisms in two nuclear receptor genes, pregnane X receptor (*PXR*) and constitutive androstane receptor *(CAR*), and the risk of ATDH in a Chinese population. Subjects with or without hepatotoxicity during anti-TB treatment were recruited. DNA was extracted from peripheral blood and genotypes of the selected single nucleotide polymorphisms (SNPs) were determined by using the improved multiplex ligation detection reaction technique. Three genetic models (additive, dominant, and recessive) as well as haplotype, SNP-SNP interaction analyses were used to evaluate the genetic risk of ATDH. A total of 502 subjects (203 ATDH and 299 non-ATDH) were enrolled. The results showed that the minor allele of rs7643645 and the H0010001 haplotype in *PXR* were associated with decreased risk of ATDH, suggesting that drug-metabolizing enzymes regulated by PXR are involved in the pathogenesis of ATDH. More studies are required to verify this result.

## Introduction

Tuberculosis (TB) remains one of the world’s biggest health problems, with 10 million new cases and approximately 1.6 million deaths in 2017 reported by WHO^1^. China had the second largest number of new TB cases in the world in 2017, with 889,000 estimated new cases and an incidence of 63/100,000^1^. Currently, 86% of newly diagnosed cases can be successfully treated, mainly due to the widespread use of standard chemotherapy (a combination of at least isoniazid, rifampicin, ethambutol and pyrazinamide for 2 months, followed by 4 months of at least isoniazid and rifampicin) in drug sensitive patients^1^. However, 15% of patients receiving treatment with these first line anti-TB drugs developed adverse drug reactions, resulting in suspension or discontinuation of one or more anti-TB drugs^2^, thereby increasing the risk of TB relapse, drug resistance, and even TB–related death^3^. Hepatotoxicity is a common and sometimes severe adverse drug reaction that occurs during anti-TB treatment, with an incidence of 2–28%, depending on the definition of hepatotoxicity and the specific population surveyed^4^.

There are four possible pathogenic mechanisms of drug-induced liver injury including the generation of reactive metabolites, oxidative stress, mitochondrial dysfunction and immunological response^5^. Unfortunately, the exact mechanisms of ATDH are not yet fully understood but multiple risk factors have been reported to associate with ATDH including genetic factors. Both environmental factors and genetic factors, as well as their interaction have been reported to be associated with ATDH^6^. Howerver, results from different study were inconsistent^6^. In addition, some studies indicated gene-gene and gene-environmental interaction may account for some variant susceptibility to ATDH in certain population^7,8^.

Genetic variants in drug metabolizing enzymes such as N-acetyltransferase 2 (*NAT2*), cytochrome P450 2E1 (*CYP2E1*) and glutathione S-transferases (*GSTM1, GSTT1*), have been the most widely studied in respect to ATDH susceptibility in the Chinese population^9–11^, as well as other populations^12^. However, these genetic biomarkers have shown poor reproducibility in some studies. Other genetic predictors of ATDH were implicated in certain populations, e.g. ATP binding cassette subfamily B member 1 in Brazilians or human leukocyte antigen in Indians^13,14^. In our previous study, we observed cytochrome P450 2B6 variants may associate with susceptibility to ATDH but only in males^15^. However, the findings to date may only represent a small minority of the genetic variants relevant to ATDH and a more reliable biomarker is needed for predicting ATDH. Genetic variants influencing the expression and/or activities of target proteins may result in a varying degree of accumulation of hepatotoxins in TB drug metabolism. However, ATDH remains unpredictable even when genetic and environmental factors are taken into account^16^. Therefore, we increased the sample size of our previous study to explore other candidate genes in addition to the phase I, II or III drug-metabolizing enzymes that were the focus of most previous studies.

The nuclear receptor subfamily 1 group I member 2 (*NR1I2*) gene, also known as pregnane X receptor (*PXR*), was first identified in 1988 as a member of the nuclear receptor superfamily, and is primarily expressed in the liver, intestine and kidney^17^. When activated by an agonist such as rifampicin, *PXR* up-regulates a large number of target drug-metabolizing enzyme genes including the cytochrome P450 family, carboxylesterases, glutathione S-transferases, UDP-glucuronosyltransferase, etc^18^, resulting in enhanced drug metabolism. Thus, during combination treatment with multiple anti-TB drugs, *PXR* may contribute to drug-drug interactions, and lead to variability in drug metabolism and disposition. Li *et al*. found that co-administration of rifampicin and isoniazid leads to higher levels of alanine aminotransferase (ALT), alkaline phosphatase, and bile plugs through a *PXR*-mediated minor metabolic pathway in a humanized *PXR* mouse model^19^. Moreover, rifampicin-mediated *PXR* activation can function as a negative regulator of inflammation and immunity, linking drug and xenobiotic metabolism to immune responses, through inhibition of the NF-κB signaling pathway^20,21^. In addition, a heightened sensitivity to oxidative toxicants was noted in both cell and mouse models upon *PXR* activation^22^. With improvement of genetic research methodology, a large number of single nucleotide polymorphisms (SNPs) in the *PXR* gene were proven to influence the function of *PXR*^23^, as well as an individual’s susceptibility to drug-associated liver injury^24^.

The nuclear receptor subfamily 1 group I member 3 (*NR1I3*), also known as the constitutive androstane receptor (*CAR*), is highly expressed in the liver and showed an overlapping set of target genes with *PXR* in response to potentially harmful chemicals^25,26^. It has been demonstrated that *CAR* gene polymorphisms may play a role in the inter-individual variability in the expression of many drug-metabolizing enzymes and in the susceptibility to drug-induced hepatotoxicity^27^. Therefore, the aim of present study was to investigate a possible correlation between *PXR* and *CAR* gene polymorphisms, together with the interaction of SNP-SNP, and risk of ATDH in Chinese TB patients.

## Results

### Patient characteristics

A total of 502 TB patients were enrolled in our study, including 203 ATDH and 299 non-ATDH. There were no statistically significant differences in the age, gender, ethnicity, height, weight, smoking, alcohol abuse and positive hepatitis B surface antigen (HbsAg) results between the patients with hepatotoxicity and those without hepatotoxicity (see Table 1 for details).

**Table 1:** Clinical and demographic characteristics of the cases and controls.

	ATDH group (n = 203)	No-ATDH group (n = 299)	P value
Age, years^†^	38.58 ± 16.42	38.42 ± 16.81	0.913^$^
Age, years^#^	36, 25.5, 68	35, 26, 71	
Female	108 (53.2)	146 (48.8)	0.336^&^
Ethnicity^‡^			0.072^*,§^
Han	181 (89.2)	280 (93.6)	
Tibetan	13 (6.4)	13 (4.3)	
Yi	7 (3.4)	6 (2.0)	
Others	2 (1.0)	0 (0)	
Smoker^‡^	57 (29.2)	77 (26.4)	0.489^§^
Alcohol abuse^‡^	25 (12.8)	33 (11.4)	0.641^§^
Height, centimeter^†^	162.85 ± 7.42	164.98 ± 7.97	0.125^$^
Weight, kilogram^†^	54.78 ± 9.89	54.94 ± 10.30	0.866^$^
Positive HBsAg^‡^	18 (9.4)	15 (5.0)	0.059^§^

^†^Data shown as mean ± standard deviation; ^#^data shown as median, interquartile range, range; ^‡^data shown as number of cases (frequency) ^§^analyzed by chi-square test; ^$^analyzed by independent sample *t* test. *P value was calculated between the Han and the other populations.

ATDH: antituberculosis drug-induced hepatotoxicity.

The severity and timing of ATDH onset are presented in Table 2. Among the ATDH group, 25.6%, 38.4% and 17.2% presented grade 1, grade 2 and grade 3 hepatotoxicity, respectively and 18.7% developed severe liver injury with ALT >10 upper limit of normal (ULN). About half of the cases of ATDH occurred in the first month of anti-tuberculosis treatment (see Table 2 for details).

**Table 2:** Frequency of hepatotoxicity according to severity and onset of antituberculosis drug-induced hepatotoxicity.

	N (total = 203)	Proportion
Severity of hepatotoxicity^†^		
Grade 1 (ALT 2–2.5 times ULN)	52	25.6%
Grade 2 (ALT 2.5–5 times ULN)	78	38.4%
Grade 3 (ALT 5–10 times ULN)	35	17.2%
Grade 4 (ALT > 10 × ULN)	38	18.7%
Onset of hepatotoxicity after antituberculosis drug usage		
<30 days	104	51.2%
30–60 days	60	29.6%
60–90 days	14	6.9%
>90 days	25	12.3%

^†^Severity of hepatotoxicity is classified according to the WHO Toxicity Classification Standards. The criterion for grade 1 was modulated to 2–2.5 times ULN in this study.

ULN: upper limit of normal.

### Association of *PXR* and *CAR* genetic polymorphisms with the risk of ATDH

Relevant features of the selected SNPs of *PXR* and *CAR* are shown in Table S1. The concordance rate of genotype results for the blind repeated samples was 99.5%. Genotype distributions of the SNPs in the ATDH and non-ATDH groups are presented in Table 3, and all the SNPs conformed to Hardy-Weinberg equilibrium (HWE) (p > 0.05). As shown in Table 3, the rs7643645 SNP in *PXR* showed statistical significance (additive model: OR = 0.704, 95% CI 0.539–0.918, P = 0.010; Dominant model: OR = 0.609, 95% CI 0.405–0.917, P = 0.017). Compared with the major allele, carrying the minor (G) allele was significantly associated with decreased ATDH risk. We also observed that the proportion of rs7643645 allele G decreased as the severity of ATDH increased (trend P = 0.003): 50.5% in controls, 43.8% in grade 1 and 2 groups, and 38% in grade 3 and 4 groups. However, we did not detect any association between the other 12 SNPs and risk of ATDH (Table 3).

**Table 3:** Association analysis between the *PXR* and *CAR* SNPs and antituberculosis-drug induced hepatotoxicity.

SNP	Genotype^†^, n case/control			P_HWE_^¶^	Additive*		Dominant*		Recessive*		
	0/0	0/1	1/1		OR (95% CI)	P	OR (95% CI)	P	OR (95% CI)		P
*PXR*											
rs7643645	70/73	97/150	36/76	0.785	0.704 (0.539–0.918)	**0.010**	0.609 (0.405–0.917)	**0.017**		0.645 (0.405–1.026)	0.064
rs6785049	65/104	107/137	31/58	0.954	0.972 (0.743–1.271)	0.833	1.208 (0.809–1.804)	0.356		0.677 (0.408–1.123)	0.131
rs3732357	88/131	97/139	18/29	0.143	1.012 (0.756–1.355)	0.936	1.030 (0.706–1.503)	0.878		0.973 (0.510–1.855)	0.934
rs3814055	117/174	71/107	15/18	0.416	1.112 (0.823–1.502)	0.489	1.068 (0.732–1.559)	0.731		1.473 (0.707–3.066)	0.301
rs2472682	71/100	102/149	30/50	0.447	0.950 (0.723–1.248)	0.713	1.001 (0.674–1.487)	0.995		0.835 (0.499–1.395)	0.490
rs3814057	45/74	110/145	48/80	0.716	1.005 (0.770–1.312)	0.970	1.299 (0.831–2.030)	0.251		0.788 (0.509–1.221)	0.286
rs2472677	82/133	88/142	33/44	0.230	0.968 (0.740–1.266)	0.811	0.891 (0.607–1.306)	0.552		1.097 (0.651–1.847)	0.729
*CAR*											
rs6686001	136/194	57/98	10/7	0.818	0.991 (0.704–1.395)	0.958	0.912 (0.614–1.354)	0.648		1.766 (0.619–5.034)	0.288
rs2307424	59/81	94/150	50/68	0.560	0.937 (0.719–1.221)	0.628	0.884 (0.584–1.337)	0.559		0.957 (0.610–1.502)	0.848
rs4073054	159/243	41/54	3/2	0.815	1.160 (0.752–1.789)	0.502	1.149 (0.723–1.823)	0.556		1.753 (0.243–12.64)	0.578
rs3003596	59/90	96/147	48/62	0.560	1.032 (0.788–1.351)	0.818	0.997 (0.661–1.504)	0.989		1.104 (0.692–1.763)	0.677
rs7530560	75/99	89/146	39/54	0.386	0.986 (0.754–1.289)	0.919	0.917 (0.616–1.366)	0.670		1.086 (0.670–1.760)	0.739
rs2502805	94/129	82/127	27/43	0.066	0.975 (0.743–1.279)	0.855	0.979 (0.668–1.434)	0.913		0.943 (0.547–1.627)	0.833

^†^“0” represents the major allele, “1” represents the minor allele.

^¶^HWE: Hardy-Weinberg equilibrium. HWE was assessed by the χ^2^ goodness-of-fit test based on the genotype distributions in this study.

*Adjusted for ethnicity, age, gender, height, weight, smoking, drinking, and HbsAg status. Additive: minor allele homozygotes versus heterozygotes versus major allele homozygotes. Dominant: heterozygotes plus minor allele homozygotes versus major allele homozygotes. Recessive: minor allele homozygotes versus major allele homozygotes plus heterozygotes.

### Association of the haplotypes of *PXR* and *CAR* with risk of ATDH

**Figure 1:**
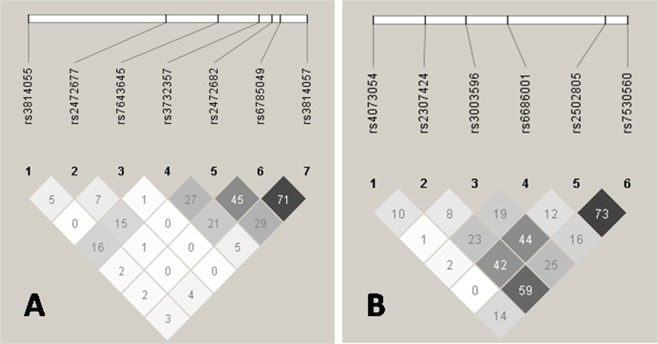
Linkage disequilibrium (LD) plots for *PXR* (**A**) and *CAR* (**B**). The LD plots were generated by Haploview 4.2. Polymorphisms are identified by their dbSNP rs numbers, and their relative positions are marked by vertical lines within the white horizontal bar. The numbers within squares indicate the r^2^ value, expressed as a percentile.

The linkage disequilibrium (LD) plots of selected *PXR* and *CAR* SNPs in the study population are shown in Fig. 1. Low LD among these SNPs was observed in both genes, except moderate LD between rs6785049 and rs3814057 in *PXR*, and between rs2502805 and rs7530560 in *CAR*. By haplotype analyses of these tagSNPs, we found 9 haplotypes in *PXR* and 6 haplotypes in *CAR* with a frequency more than 0.03 (Tables 4 and 5, respectively). Among these common haplotypes, one haplotype (H0010001) in the *PXR* gene had a significant association with decreased ATDH risk (OR = 0.591, 95% CI 0.377–0.927, P = 0.021).

**Table 4:** Analyses of derived haplotypes (frequency more than 3%) from seven polymorphisms of *PXR* with the risk of ATDH.

Haplotypes^†^	ATDH group		Non-ATDH group		OR	95% CI	P value
	n	%	n	%			
H0000001	24	5.9	26	4.4	1.362	0.767–2.419	0.291
H0010001	31	7.7	71	11.9	0.591	0.377–0.927	0.021
H0010110	36	9.0	50	10.5	1.069	0.678–1.686	0.774
H0100110	34	8.4	59	9.9	0.806	0.514–1.263	0.346
H0101000	21	5.2	23	3.8	1.280	0.688–2.383	0.435
H0101001	16	3.9	25	4.2	0.839	0.466–1.858	0.931
H0110110	27	6.7	31	5.1	1.315	0.768–2.252	0.318
H1101001	43	10.5	51	8.5	1.138	0.730–1.776	0.568
H1111001	16	3.9	31	5.2	0.730	0.391–1.360	0.320

^†^“0” represents the major alleles, “1” represents the minor alleles.

Order of polymorphisms: rs3814055, rs2472677, rs7643645, rs3732357, rs2472682, rs6785049, rs3814057. ATDH: antituberculosis drug-induced hepatotoxicity.

**Table 5:** Analyses of derived haplotypes (frequency more than 3%) from six examined polymorphisms of *CAR* with the risk of ATDH.

Haplotypes^†^	ATDH group		Non-ATDH group		OR	95% CI	P value
	n	%	n	%			
H001000	114	28.0	169	28.4	0.946	0.714–1.254	0.699
H011000	47	11.7	57	9.6	1.210	0.804–1.819	0.360
H010011	115	28.2	182	30.5	0.863	0.653–1.141	0.300
H111001	28	6.9	33	5.5	1.239	0.735–2.089	0.421
H000100	76	19.0	107	17.9	1.040	0.751–1.440	0.815
H110011	18	4.5	23	3.9	1.139	0.607–2.135	0.686

^†^“0” represents the major alleles, “1” represents the minor alleles.

Order of polymorphisms: rs4073054, rs2307424, rs3003596, rs6686001, rs2502805, rs7530560. ATDH: antituberculosis drug-induced hepatotoxicity.

### SNP-SNP interactions with risk of ATDH

We carried out a multifactor dimensionality reduction (MDR) analysis with all the tested SNPs to investigate potential genetic interactions associated with ATDH. We limited the interaction models from two-way to five-way. However, we did not identify a multilocus model with receivable cross-validation consistency (from 2/10–5/10). Moreover, all these models did not reach the threshold value of statistical significance (Table S2).

### Power analysis

We calculated the power of the sample size for the 13 selected SNPs under the allelic models (Figs S1 and S2). The results showed that our study has reasonable power (>90%) to draw conclusions with OR1.6 or above, except rs4073054 which has a low MAF of 0.105.

## Discussion

ATDH remains a serious problem for TB prevention and treatment due to the limited understanding of its pathogenesis. Therefore, biomarkers which can detect patients with different susceptibility to ATDH are urgently needed to further understanding of the pathogenesis of this adverse event and for guiding clinical decision making^28^. In the present association study, we focused on the genetic polymorphisms of the nuclear receptor, which can influence a wide range of metabolizing enzymes in the liver that are involved in drug-drug interactions^29^. We found that rs7643645 and a haplotype of the *PXR* gene were associated with ATDH. The *PXR* rs7643645 G allele and individuals carrying the H0010001 haplotype showed significantly decreased risk for ATDH.

Genetic polymorphisms of *NAT2*, *CYP2E1* and the *GST* family are the most studied in relation to ATDH, for they may affect the metabolism and elimination of isoniazid, or are involved in oxidant-antioxidant balance in liver cells after exposure to anti-TB drugs^12^. We previously demonstrated that *CYP2E1, GSTP1* and *CYP2B6* were associated with susceptibility to ATDH by a meta-analysis and a case-control study^15,30,31^. Moreover, NAT2 mediates isoniazid biotransformation to form acetylhydrazine, which then undergoes oxidization by CYP2E1 to form a hepatotoxic substance^32^, but there is little evidence to implicate these enzymes in the metabolism of other anti-TB drugs such as pyrazinamide, which account for approximately 60% of ATDH^33^. The incidence of liver injury was higher in patients with co-treatment of rifampicin and isoniazid than taking either drug alone^34^. A widely accepted explanation for this is based on the rifampicin-mediated up-regulation of metabolic enzymes, which may contribute to isoniazid bioactivation and toxicity^35,36^. *PXR* serves as the main acceptor of rifampicin, and may be involved in this drug-drug interaction and the pathogenesis of ATDH. A previous study suggested that multiple SNPs, including rs7643645, in the *PXR* gene influenced the expression of *PXR* and its target genes in donor livers with or without rifampicin pretreatment^23^. The G allele of rs7643645 showed loss of a potential hepatic nuclear factor 4α binding site and was associated with lower *CYP3A*, *MDR1* and *PXR* mRNA expression, compared to the A allele^37,38^. The lower level of drug-drug interaction in individuals carrying the *PXR* rs7643645 GG genotype may be a potential protective mechanism, which is consistent with our result. Another potential pathogenesis of ATDH is the imbalance between production of reactive oxygen species and detoxification of reactive intermediates^39^. Gong *et al*. found that mice treated with a *PXR* agonist and cell lines with activated human *PXR* showed heightened sensitivity to oxidative toxicants, with an increased production of reactive oxygen species^22^. Therefore, further study is needed to determine whether the rs7643645 G allele confers lower *PXR* activity and expression, and reduced susceptibility to oxidative stress and ATDH.

Surprisingly, a previous study by Wang *et al*. concluded that the rs7643645 AA genotype was associated with the decreased risk of ATDH in females^40^. However, a large number of studies indicate that females have a higher risk of ATDH than males^41,42^. *CYP3A* activity was higher in females compared with males^43^. We reasoned that the rs7643645 GG genotype with lower *CYP3A* activity may lead to decreased risk of ATDH. This hypothesis should be tested in future studies. Moreover, we excluded the potential risk factors of ATDH such as cancer and renal failure, which made up nearly 30% of the subjects by Wang *et al*. study and this may make our results more reliable. However, some differences between two studies should also be taken into consideration, e.g. the study design (Wang *et al*. performed a prospectively conducted study^40^, while we utilized a case-control study) and the definition of ATDH (Wang *et al*. defined ATDH as ALT > 5 × ULN in asymptomatic patients and ALT > 3 × ULN in symptomatic patients^40^, while we used criteria recommended by an international consensus meeting which are described in the methods section). Another genetic association study by Zazuli *et al*. demonstrated that *PXR* rs3814055 was associated with risk of ATDH^44^, which is inconsistent with our result. We believe these conflicting findings may be due to differences in ethnicity and definition of ATDH between the studies. In the study by Zazuli *et al*., ATDH was designated as an increase in serum ALT and AST levels above the ULN. Moreover, if we raised the ALT threshold (ALT > 5 ULN) for ATDH, that recommended by the Chinese Society of Hepatology in 2015^45^, to further explore the clinical utility of testing for rs7643645, we observed a stable positive result (cases: 73, controls 299; additive model: OR = 0.601, 95% CI = 0.416–0.817, P = 0.006; dominant model: OR = 0.400, 95% CI = 0.190–0.842, P = 0.013; recessive model: OR = 0.574, 95% CI = 0.335–0.986, P = 0.043).

We did not find any positive results for the SNPs in the *CAR* genetic region. There are several reasons to explain this lack of association. Firstly, although the sample size of our study gave an acceptable power (>90%) to detect a common risk allele with an OR of 1.6 in most SNPs, the analysis of rs4073054 was still underpowered (Figs S1 and S2). Secondly, antituberculosis drugs and their metabolites were metabolized by enzymes such as NAT2 or CYP2E1^28^. *CAR* or its genetic variation may not play a pivotal role in this specific pathway. Given that combined analyses of SNPs may display a more complete picture of the candidate genes, we further conducted a haplotype analysis and, a SNP-SNP interaction analysis of the selected tagSNPs. We only found that a haplotype in *PXR* (H0010001) showed decreased risk of ATDH.

There are several strengths of our study. We limited the confounding factors by excluding the comorbidities of HIV infection, cancer, renal failure or cardiac dysfunction and estimated the association between genetic factors and risk of ATDH with adjustment for other confounders. Moreover, in order to avoid potential bias, those who were in charge of clinical data and those who were responsible for laboratory data worked independently in this study. In addition, SNPs in the Chinese Beijing Han population located between 3000 bp upstream and 300 bp downstream of each gene were systematically reviewed to maximize inclusion of functional SNPs. Although some non-Han subjects were included in this study (i.e. 26 Tibetan, 13 Yi, and 2 others), a previous genome-wide association study found that Chinese Han and Tibetans share a similar genetic background^46^. There is a paucity of similar studies for Yi and other Chinese populations. Therefore, we had no alternative but to choose the SNP information of the Beijing Han population. We believe that the small proportion of Yi and other groups (3%) had only a minor effect on our results. If the subgroup of Chinese Han only was analyzed, we found rs7643645 was still significantly associated with decreased risk of ATHD (cases: 181, control: 280; additive model: OR = 0.607, 95% CI = 0.461–0.800, P < 0.001; dominant model: OR = 0.504, 95% CI = 0.334–0.760, P = 0.001; recessive model: OR = 0.539, 95% CI = 0.332–0.875, P = 0.012).

Nevertheless, some potential limitations should be taken into consideration when interpreting our results. Firstly, the activity of enzymes including *PXR*, *CAR* and other related downstream metabolic enzymes were not evaluated in the individuals. Secondly, a large number of previous studies have shown that polymorphisms of multiple genes may be involved in ATDH, suggesting the occurrence of ATDH may result from the combination of variation in several susceptibility genes, such as *NAT2* or *CYP2E1*^47^. However, we did not genotype other candidate genes. Thirdly, in this retrospective case-control study, there were some inevitable biases in the characteristics of the patients. We limited these biases by using a combination of face-to-face questionnaires and screening the medical records, and excluded the subjects with incomplete or uncertain clinical/demographic data. In addition to HBV co-infection, alcohol consumption, and smoking (which we used as covariates in the analysis), hepatitis C virus (HCV) co-infection was also shown to be a risk factor for ATDH^48^. However, according to an epidemiologic study with a total of 236,920 individuals performed in China^49^, the incidence of HCV co-infection is just 3%. Therefore, we did not test for HCV co-infection in all the participants. Despite this, we searched the medical records in West China Hospital and found that 214 patients (96 in ATDH group and 118 in no-ATDH group) had been serologically tested for anti-HCV. The incidence of HCV co-infection was 3.1% (3/96) in ATDH group and 1.7% (2/118) in the no-ATDH group. Therefore, this risk factor may only have caused a minimal bias. We also analyzed the subjects without HBV/HCV co-infection, and found the result did not change.

In conclusion, *PXR* genetic polymorphism may be a valuable biomarker potentially involved in ATDH. More studies are required to verify our results.

## Methods

### Subjects

Ethical approval for this study was obtained from the Institutional Review Board of the West China Hospital of Sichuan University. Methods were carried out in accordance with the approved guidelines. Written informed consent was obtained from each subject. In this case-control study, we recruited TB patients who were diagnosed and treated in the West China Hospital, Chengdu, Sichuan Province, People’s Republic of China between October 2013 and October 2016. The inclusion criteria for the control group (non-ATDH group) were as follows: (a) Normal serum ALT, aspartate aminotransferase and bilirubin levels before anti-TB treatment. (b) Daily treatment with isoniazid, rifampicin, ethambutol and pyrazinamide for at least 2 months, followed by daily treatment with isoniazid, rifampicin and ethambutol, with the total course of treatment equal to or more than 6 months. The dosage of each drug was as follows: isoniazid 300 mg/d, rifampicin 450 mg/d ≤ 50 kg body weight and 600 mg/d > 50 kg body weight, ethambutol 750 mg/d, pyrazinamide 1500 mg/d. (c) At least monthly liver function tests and all with normal results. The inclusion criteria for the case group (ATDH group) were as follows: (a) Normal serum ALT, AST and bilirubin levels before anti-TB treatment. (b) During the period of first line anti-TB treatment (the dosage of each drug was the same as the no-ATDH group), patients meeting the criteria of ATDH reported by an international consensus meeting 1990^50^. In detail, the definition of ATDH was at least one instance of ALT greater than 2 times the ULN and/or a combined increase in AST and total bilirubin, provided one of them was greater than 2 times the ULN, as previously defined. The cutoff of ULN was 50 IU/L for ALT and AST, 28 umol/L for total bilirubin. (c) No administration of other potentially hepatotoxic drugs in the two weeks before ATDH. (d) Liver function tests returned to normal after suspension of anti-TB drugs. Exclusion criteria were: a) Refusal to provide a blood sample or to sign an informed consent form. (b) Positivity for HIV/AIDS. (c) Pregnancy. (d) Cancer, renal failure or cardiac dysfunction that may cause liver dysfunction. (e) Incomplete clinical/demographic data. (f) age <14 year old. The definition of alcohol abuse was daily consumption of more than 60 g of ethanol per day which is based on the WHO criteria (Global Status Report on Alcohol and Health 2014, http://apps.who.int/iris/bitstream/10665/112736/1/9789240692763_eng.pdf?ua=1).

### Sample collection and processing

After obtaining consent, we collected a 5 ml peripheral blood specimen from each patient in ethylene diamine tetraacetic acid coated tubes (BD Vacutainers, Franklin Lakes, NJ, USA). The plasma was isolated within 24 hours for determination of the HBsAg by commercial enzyme-linked immunosorbent assay kits (Abbott Park, Wiesbaden, Germany) and the rest of blood specimen was used for extraction of genomic DNA by commercial kit (Axygen Scientific Inc, Union City, CA, USA). The DNA samples were stored at −80 °C until further analysis.

### Selection and genotyping of tagging SNPs

All eligible SNPs within 3000 bp upstream and 300 bp downstream of the *PXR* and *CAR* genes in the Chinese Han Beijing population were obtained from the HapMap database (Phase II + III Release 27; NCBIB 36; www.hapmap.org). All the SNPs in these regions were filtered using a pairwise tagging algorithm by the Haploview v4.2 software program^51^, with a minor allele frequency (MAF) ≥0.1 and a LD r^2^ measure ≥0.80. The LD plots of SNPs based on the data from the HapMap Project in the *PXR* and *CAR* genes are shown in Figs S3 and S4, respectively. After calculation by Haploview v4.2, we obtained 7 blocks and 6 blocks for *PXR* and *CAR*, respectively. The tagSNPs were selected from each block based on 3 principles. (a) Evidence from the literature that the SNP alters the expression or activity of *PXR*, *CAR* or its targets. (b) Association with drug-induced liver injury in a previous study. (c) Functional SNPs predicted by FastSNP^52^.

Genotyping of the selected SNPs was performed by laboratory technicians who were blind to the clinical data, using an improved multiplex ligation detection reaction (iMLDR) technique developed by Genesky Biotechnologies Inc. (Shanghai, China)^53^. We also used 10% blind repeated samples for quality control of genotyping. The primer and probe information is shown in Table S3. Raw data were analyzed using the GeneMapper 4.1 software program (Department of Computer Science, University of California at Berkeley, Berkeley, CA, 94720, USA).

### Statistical analysis

Statistical analyses were performed using SPSS version 17.0 (Chicago, Illinois, USA). All tagSNPs were tested for agreement with HWE by a goodness-of-fit χ^2^ test. Quantitative variables were expressed as mean ± standard deviation (SD), and qualitative variables were presented as percentages. The demographic and clinical data of the ATDH group and the no-ATDH group were compared using the χ^2^ test and Student’s t-test. Three genetic models (additive, dominant, and recessive) were used for assessing the genotype risk of ATDH. Association between genotypes and ATDH risk was evaluated by P value and odds ratio (OR) as well as corresponding 95% confidence intervals (CI) by logistic regression. Covariates considered for inclusion in the regression analysis were ethnicity, age, gender, height, weight, smoking, drinking, and HbsAg result. LD among the selected SNPs was assessed by Haploview v4.2 (http://www.broad.mit.edu/mpg/haploview/)^51^. SHEsis online software was applied for the haplotype analysis of *PXR* and *CAR* gene polymorphisms (http://analysis.bio-x.cn/myAnalysis.php)^54^. Rare haplotypes with a frequency less than 0.03 were collapsed into one category in the final haplotype analyses. Two-sided values of p < 0.05 were considered statistically significance. Furthermore, we used the Multifactor Dimensionality Reduction Software (version 3.0.2) to analyze the SNP-SNP interactions associated with ATDH^55^. An overall best model was selected that had high cross-validation consistency and maximum testing balanced accuracy. P < 0.05 by comparing the observed average CVC of each chosen model with the distribution of average consistencies under the null hypothesis of no associations derived empirically from 1000 permutations was considered significant. We also calculated the power of our study design using Power and Simple Size Calculation Software (http://biostat.mc.vanderbilt.edu/PowerSampleSize).

## Supplementary information

Supplementary Materials

Table S1

Table S2

Table S3
