# Supplementary material for: Association of PXR and CAR Polymorphisms and Antituberculosis Drug-Induced Hepatotoxicity
Source: Sci Rep. 2019 Feb 18;9:2217. doi: 10.1038/s41598-018-38452-z (PMC6379441; doi:10.1038/s41598-018-38452-z)
Supplement: Supplementary file 1 — Supplementary Materials [file 41598_2018_38452_MOESM1_ESM.pdf]

Supplementary Materials

Association of PXR and CAR Polymorphisms and Antituberculosis Drug-Induced Hepatotoxicity

Yu Wang,[a] Xi Xiang,[a] Wei-Wei Huang,[a] Andrew J Sandford,[b] Shou-Quan Wu,[a] Miao-Miao Zhang,[a] Ming-Gui Wang,[a] Guo Chen,[a,c] Jian-Qing He[a*]

[a]Department of Respiratory and Critical Care Medicine, West China Hospital, Sichuan University, Chengdu, Sichuan, China

[b]Centre for Heart Lung Innovation, University of British Columbia and St. Paul's Hospital, Vancouver, BC, Canada

[c]Division of Geriatrics, Sichuan Provincial People's Hospital, Chengdu, Sichuan, China

Running title: PXR, CAR polymorphisms and risk of ATDH

[*]Corresponding author: Dr. Jian-Qing He

Address: Department of Respiratory and Critical Care Medicine, West China Hospital, Sichuan University, No. 37 Guo-xue alley, Chengdu 610041, Sichuan Province, China.

Tel:01186-18980602293; Fax: 01186-85422571; E-mail: jianqhe@gmail.com

Y.W., X.X., W.W.H. contributed equally to this work.

Legends

Table S1: Characteristics of the SNPs in this study.

Table S2: SNP-SNP interaction analyzed with MDR.

Table S3: Primer and probe information for the 13 tagSNPs detected by improved multiplex ligation detection reaction (iMLDR).

Fig. S1: Statistical power of each SNP in PXR. The power was calculated by Power and Simple Size Calculation Software.

Fig. S2: Statistical power of each SNP in CAR. The power was calculated by Power and Simple Size Calculation Software.

Fig. S3: Linkage disequilibrium (LD) plots for the SNPs between 3000bp upstream and 300bp downstream of PXR based on the Chinese Beijing Han HapMap population. (A) The D' LD measure and (B) the r2 LD measurement. The strength of the LD between SNPs is indicated by the color scheme, measured using D' and r2 statistics.

Fig. S4: Linkage disequilibrium (LD) plots for the SNPs between 3000bp upstream and 300bp downstream of CAR based on the Chinese Beijing Han HapMap population. (A) The D' LD measure and (B) the r2 LD measurement. The strength of the LD between SNPs is indicated by the color scheme, measured using D' and r2 statistics.

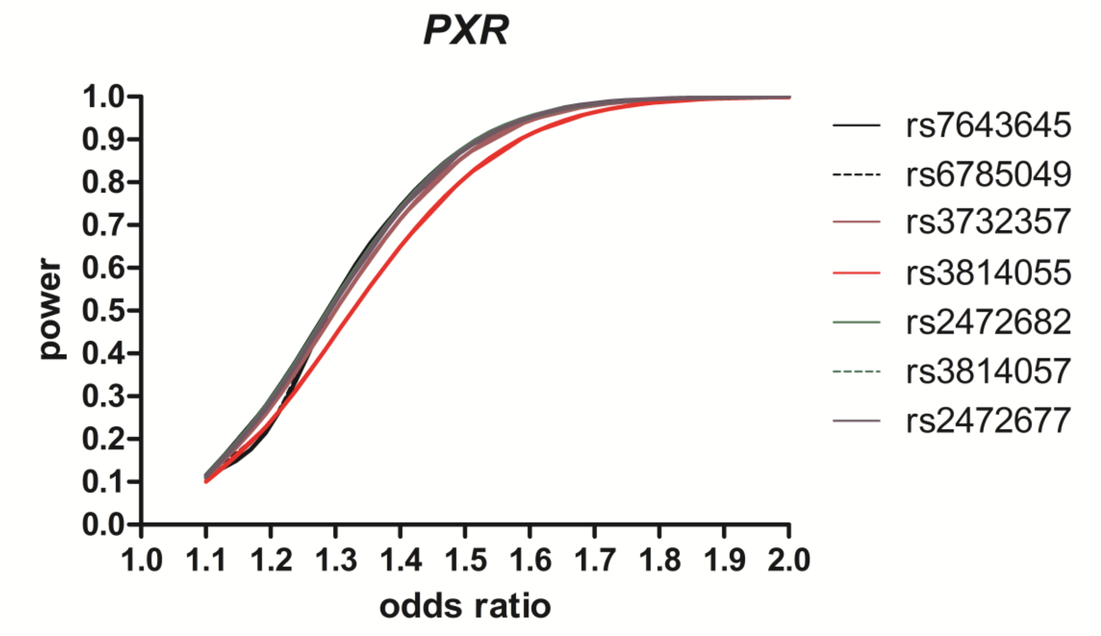

Fig. S1: Statistical power of each SNP in *PXR*. The power was calculated by Power and Simple Size Calculation Software.

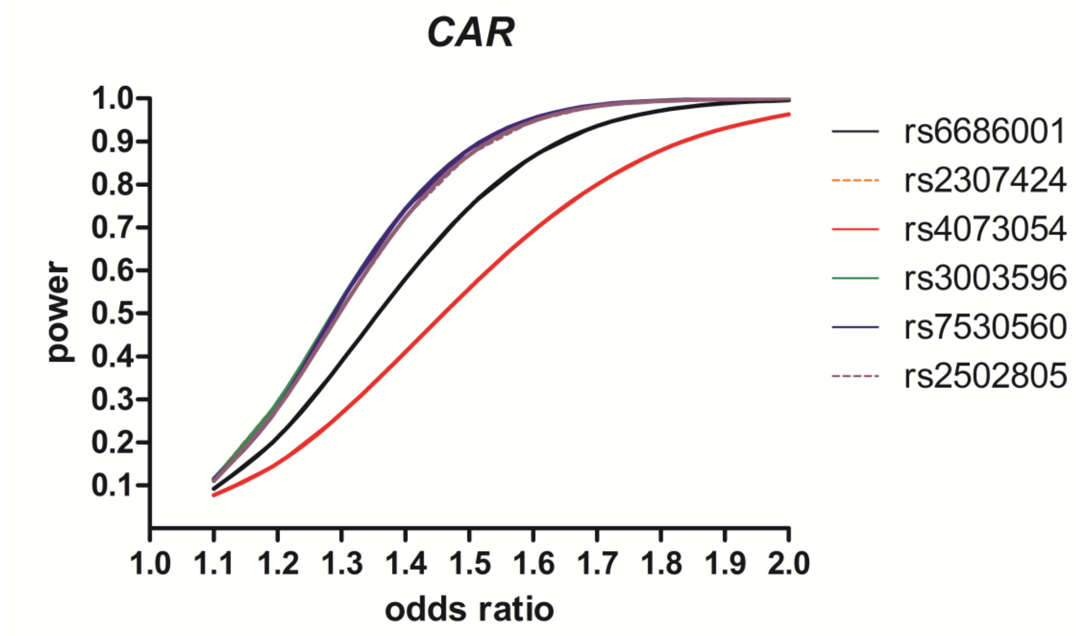

Figure S2: Statistical power of each SNP in *CAR*. The power was calculated by Power and Simple Size Calculation Software.

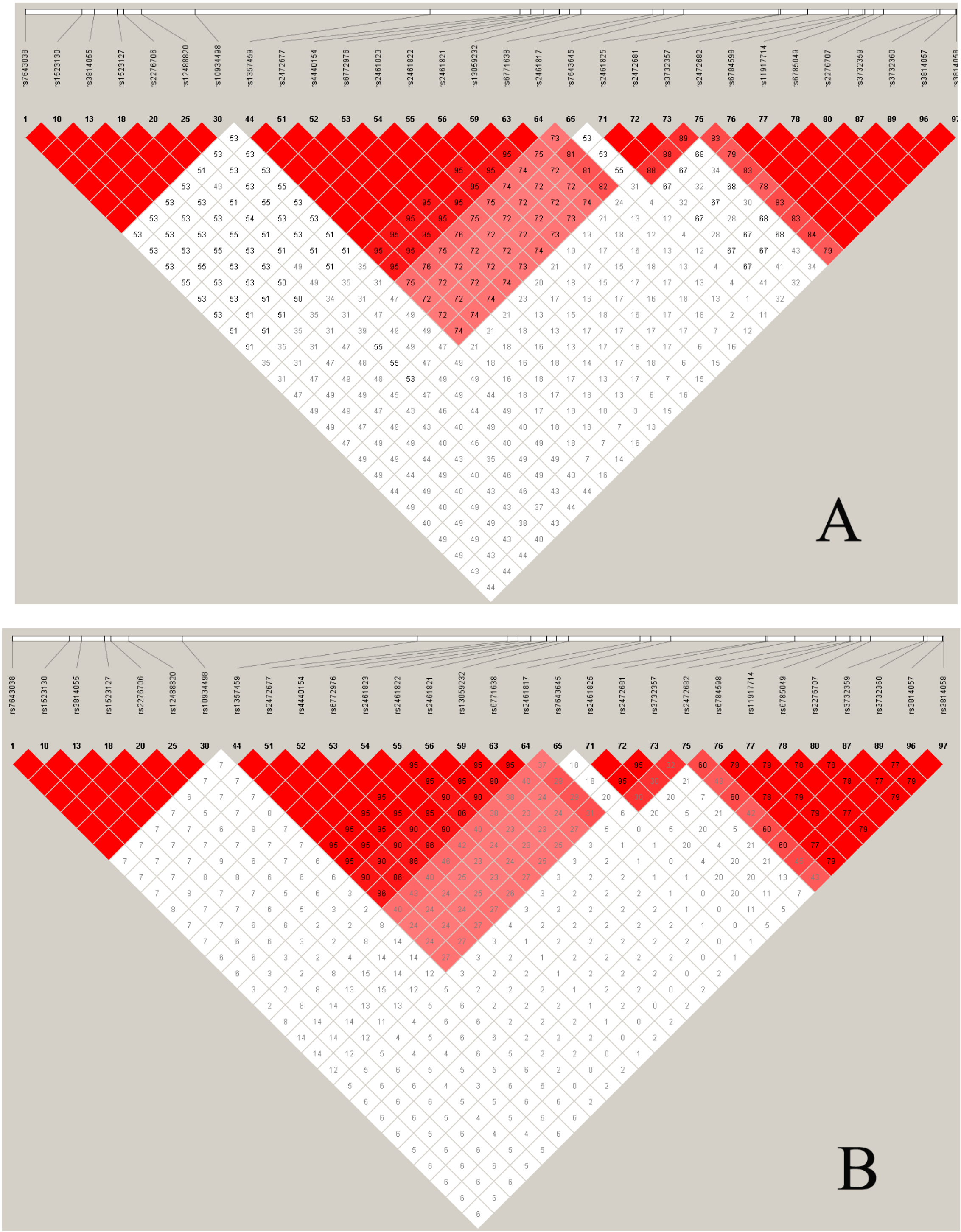

Figure S3: Linkage disequilibrium (LD) plots for the SNPs between 3000bp upstream and 300bp downstream of *PXR* based on the Chinese Beijing Han HapMap population. (A) The D' LD measure and (B) the r^2 LD measurement . The strength of the LD between SNPs is indicated by the color scheme, measured using D' and r^2 statistics.

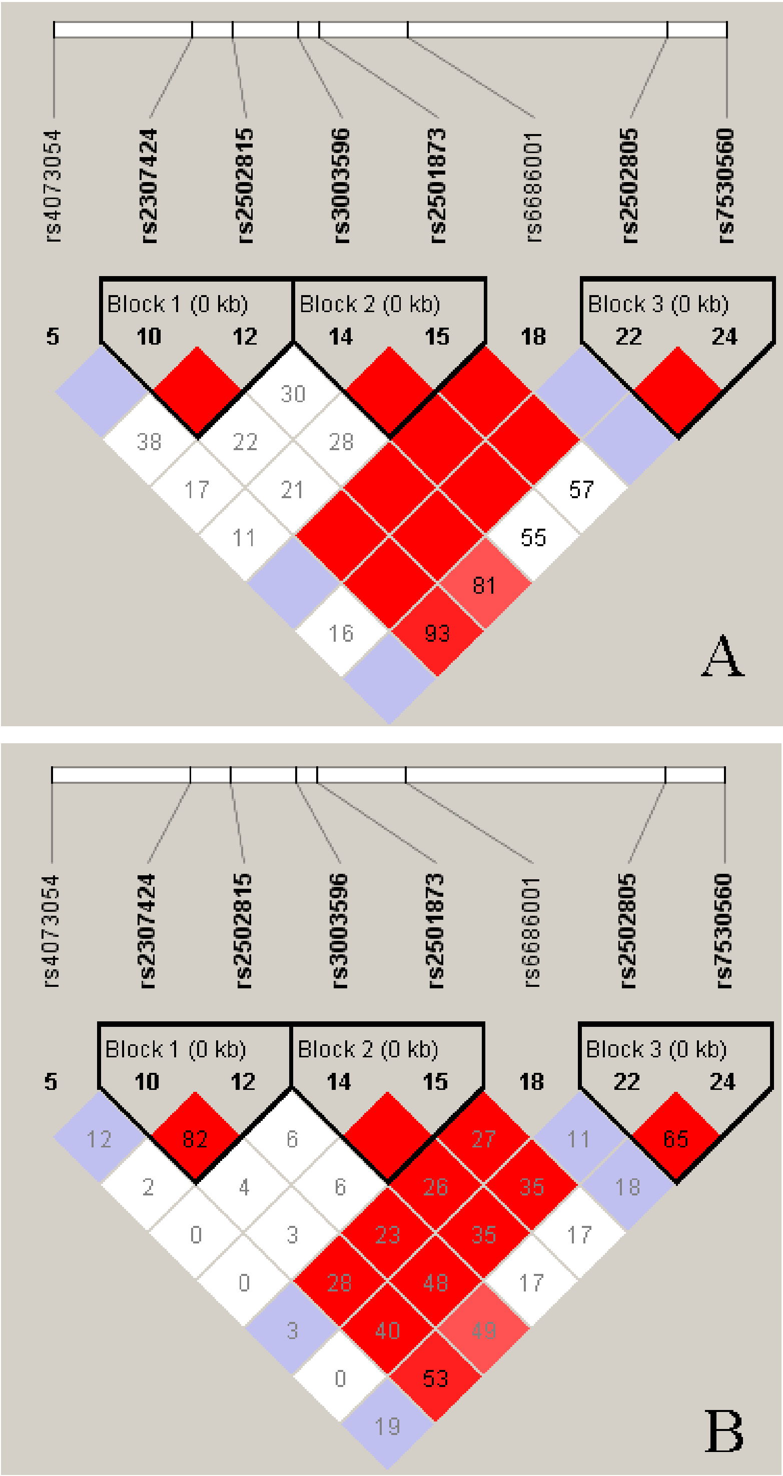

Figure S4: Linkage disequilibrium (LD) plots for the SNPs between 3000bp upstream and 300bp downstream of *CAR* based on the Chinese Beijing Han HapMap population. (A) The D' LD measure and (B) the r^2 LD measurement. The strength of the LD between SNPs is indicated by the color scheme, measured using D' and r^2 statistics.
